# Leflunomide-induced collagenous colitis: a case report and literature review

**DOI:** 10.1007/s12328-023-01862-2

**Published:** 2023-10-05

**Authors:** Jamie O. Yang, Sarah Dry, Guy A. Weiss

**Affiliations:** 1 UCLA Department of Internal Medicine, Los Angeles, CA USA; 2 UCLA Department of Pathology & Lab Sciences, Los Angeles, CA USA; 3 Vatche and Tamar Manoukian Division of Digestive Diseases, UCLA David Geffen School of Medicine, Los Angeles, CA USA

**Keywords:** Leflunomide, Colitis, Diarrhea

## Abstract

We describe a patient with rheumatoid arthritis and Hashimoto’s thyroiditis who developed chronic diarrhea and subsequently diagnosed with collagenous colitis (CC) 5 years after leflunomide initiation. Cessation of leflunomide resulted in complete resolution of diarrhea within 2 months. Although rare, leflunomide-induced colitis should be considered in patients with otherwise unexplained chronic diarrhea. Diagnosis is challenging as symptom onset can occur many years after leflunomide initiation, but diarrheal symptoms typically resolve within weeks to months of stopping the instigating drug.

## Introduction

Leflunomide is a disease-modifying anti-rheumatic drug (DMARD) that acts by inhibiting the mitochondrial enzyme dihydroorotate dehydrogenase, which prevents the synthesis of pyrimidines, effectively halting the proliferation of autoimmune T lymphocytes [1]. It has been FDA-approved for treating rheumatoid arthritis (RA) since 1998. In the leflunomide trials, an estimated 10–20% of leflunomide-treated RA patients experienced gastrointestinal symptoms, such as diarrhea, nausea, and abdominal pain [1–3]. Other common adverse reactions associated with leflunomide-included allergic reactions, infections, and elevated liver enzymes. However, these symptoms tended to be acute, peaking during the first 2 weeks of treatment, and then self-resolving after the first 6 months, not requiring discontinuation of therapy [4, 5]. Our literature review resulted in less than ten case reports of leflunomide-associated colitis (microscopic or macroscopic). We present a case of leflunomide-induced collagenous colitis occurring 5 years from leflunomide initiation, that resolved after discontinuation of leflunomide.

## Case report

**Fig. 1:**
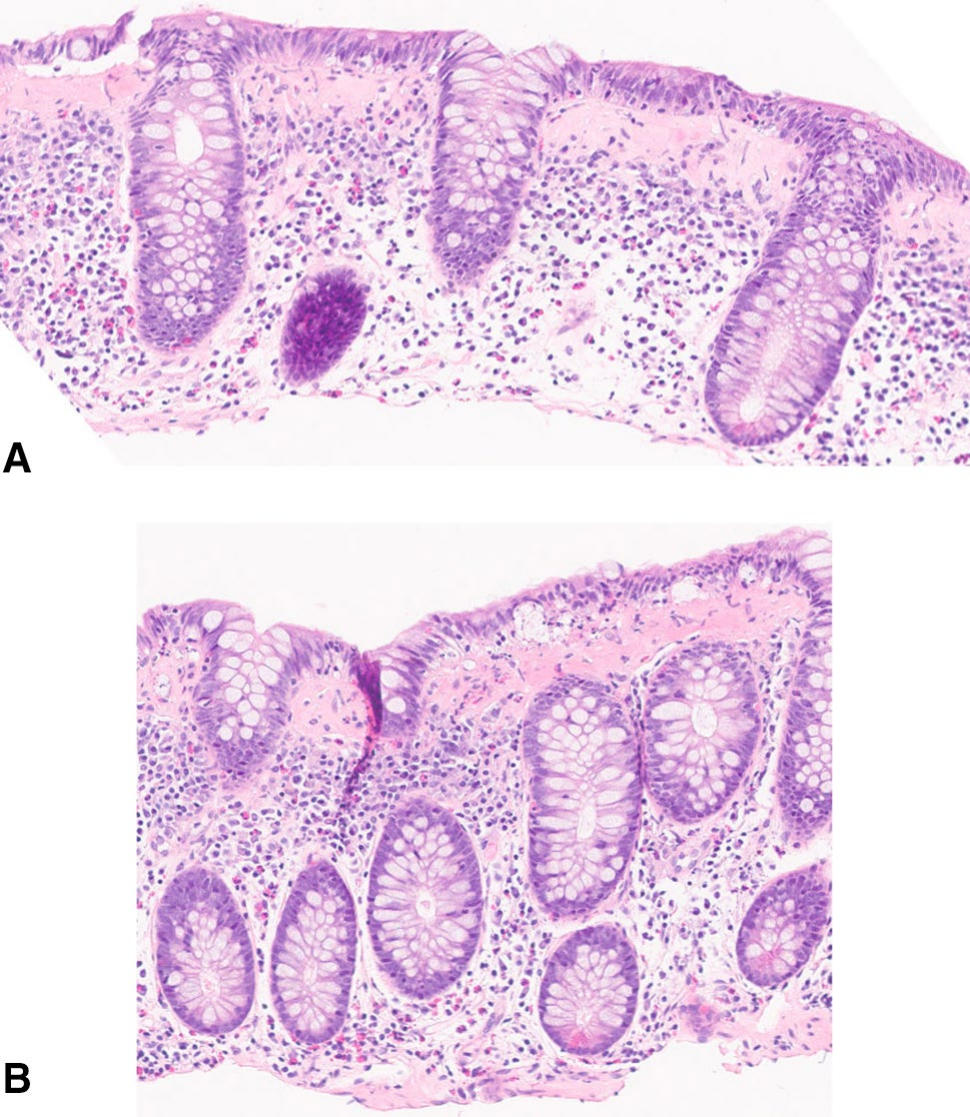
Histopathological images demonstrating collagenous colitis. **A** Transverse colon 10 × magnification. **B** Right colon 10 × magnification. Sections of the colon show a thick subepithelial collagen band and increased chronic inflammation in the superficial lamina. The superficial epithelium lifts off in places from the underlying mucosa. Chronic inflammatory cells are present in low numbers overall in the superficial epithelium, with foci that have a higher concentration of inflammatory cells

A 78-year-old woman with RA, Hashimoto’s thyroiditis, and chronic kidney disease was referred to our gastroenterology clinic for epigastric and lower abdominal pain and chronic diarrhea for 6 months. She reported a change from her baseline of 1–2 formed bowel movements daily [Bristol Stool Form Scale (BSFS) 2–3] to 4–5 loose (BSFS 7) bowel movements per day. She denied nocturnal episodes, rectal bleeding, fever, or chills. Her medications included amlodipine, duloxetine, levothyroxine, losartan, rosuvastatin, and leflunomide 25 mg daily, which she had been taking for 5 years. Workup including infectious etiologies (bacterial and parasitic enteric pathogen PCR panels), fecal calprotectin, and complete celiac disease serologies (endomysial IgA, transglutaminase IgA, and deamidated gliadin peptide IgA/IgG) were all normal. She subsequently underwent endoscopic evaluation. Colonoscopy revealed a small polyp in the transverse colon but otherwise macroscopically normal mucosa. Biopsies of the ascending, transverse, and descending colon were taken, and pathology showed a thick subepithelial collagen band with increased chronic inflammation in the superficial lamina, consistent with collagenous colitis (Fig. 1).

In consultation with her rheumatologist, her leflunomide was held. A month later, her bowel movement frequency had decreased from 4–5 to 3–4 per day, with intervals of 1–2 days without any bowel movements at all. Her other gastrointestinal symptoms of nausea and abdominal pain had improved as well. The following month, she reported complete resolution of her diarrhea. Given that her diarrhea had resolved after stopping the leflunomide, this medication was deemed to be the culprit, as opposed to duloxetine or rosuvastatin, which were continued, both of which have been also associated with microscopic colitis [6, 7]. Under the care of her rheumatologist, she was started on methotrexate and rituximab for RA with no further issues.

## Discussion

Leflunomide-induced colitis is a very rare side effect with only a few cases reported in the literature. The mechanism by which leflunomide induces colitis is not well understood. Furthermore, it seems that the presentation is greatly varied, both in types of colitis and in timeline from leflunomide initiation. Our literature review of studies published in English revealed ten cases of leflunomide-induced colitis (Table 1). Of the ten cases, four had microscopic colitis, a type of colitis that appears grossly normal on endoscopy, but inflammation can be seen through histologic evaluation. Microscopic colitis encompasses two main subtypes, collagenous colitis (CC) and lymphocytic colitis (LC), which are differentiated primarily through histology [8]. LC has an increased proportion of surface intraepithelial lymphocytes, while CC has a thickened collagenous subepithelial band [8]. It remains a point of debate whether the microscopic colitis subtypes are the same disease with different expressions, as patients have been shown to have features of both or different histologic features sampled at different time points [9]. In our literature review of leflunomide-induced microscopic colitis cases, one patient had LC, two patients demonstrated CC, and one patient had features of both LC and CC (Table 1). These cases presented with diarrhea on average 2 years after leflunomide initiation. Our patient demonstrated CC, and presented 5 years after leflunomide initiation. In patients who presented with other types of colitis, onset of diarrhea ranged anywhere from 2 months to 6 years.

**Table 1:** Summary of reported cases of leflunomide-induced colitis in the literature

Age (years)	Gender (M/F)	Time from leflunomide initiation to symptom onset	Time until symptom resolution after leflunomide discontinuation	Colonoscopy findings	Pathology-microscopic findings	References
Microscopic colitis						
63	F	2 years	Not reported	Macroscopically normal	Lymphocytic colitis and collagenous colitis	[5]
64	F	18 months	20 days	Colonic mucosal edema	Collagenous colitis- increase in intraepithelial lymphocytes and a thickened collagen layer	[10]
73	F	3 years	Not reported	Macroscopically normal	Left-sided collagenous colitis	[11]
55	F	Not reported	3 days	Macroscopically normal	Lymphocytic colitis	[12]
Other types of colitis						
39	F	18 months	Not reported	Multiple punctiform mucosal Bleeding centers throughout colon	Ulcerative and hemorrhagic colitis	[5]
61	F	6 years	Not reported	Mayo 2-3 colitis, mucosal hyperemia and ulcerations	Moderate active colitis, with cryptitis, crypt abscesses and significant apoptosis	[13]
68	M	5 years	7 weeks	Mucosal inflammation Subtle small erosions in terminal ileum and throughout colon	Active and chronic inflammation, including crypt abscesses, cryptitis, scattered granulomas within lamina propria, and lymphocyte infiltration	[14]
46	F	30 months	3 weeks	Mucosal hyperemia and multiple colonic aphthous ulcerations	Focal ulceration, cryptitis, mixed inflammation at the lamina propria	[15]
46	F	17 months	2 weeks	Circumferential, contiguous inflammation throughout colon, normal terminal ileum	Surface erosion, inflammation at the lamina propria, crypt abscesses	[16]
49	F	2 months	1 month	Deep colonic ulcers, severe colitis	Focal active colitis	[17]

In all cases including this current one, gastrointestinal symptoms such as diarrhea resolved after leflunomide cessation, which supported the diagnosis of leflunomide-induced colitis. Average time until symptom resolution after leflunomide discontinuation was about 3 weeks, but ranged from 3 days to 7 weeks. Our patient’s time to symptom resolution was around 2 months, at the upper reported duration. It is important to note that leflunomide is administered orally and metabolized in the gut into the active metabolite teriflunomide. In RA patients, the half-life of teriflunomide is approximately 15 days, and excreted primarily through the urine and feces [18]. Thus, metabolism of leflunomide can differ between patients based on their metabolism and kidney function, and likely prolonged in our patient with chronic kidney disease. Considering this long half-life and average time of 3 weeks for symptom, resolution in these reported cases further supports the association between leflunomide and colitis.

The wide variability of patient presentations makes it challenging to create definitive diagnostic criteria for leflunomide-induced colitis. Part of the limitation is due to the rarity of this condition, and more case reports on this topic are needed before we can draw conclusions about classical findings. Based on the current literature, the most common symptom in leflunomide-induced colitis is chronic diarrhea that begins years after leflunomide initiation. One limitation was that our patient was not re-challenged with leflunomide which would have helped definitively support the diagnosis. This diagnosis remains a diagnosis of exclusion, as all other etiologies for diarrhea, including infection, inflammatory, autoimmune, and endocrinological causes, should be ruled out. A careful review of a patient’s medication list should also be done, as there are other drugs that can induce colitis, most commonly nonsteroidal anti-inflammatory drugs, proton-pump inhibitors, selective serotonin reuptake inhibitors, angiotensin-converting enzyme inhibitors, beta blockers, and statins [19]. A diagnosis of leflunomide-induced colitis is supported if diarrhea improves after 3 weeks of leflunomide discontinuation, although it can take up to 2 months. Thus, in patients being treated with leflunomide who develop persistent diarrhea with no other explanation, a colonoscopy with biopsy is warranted, and discontinuation of leflunomide should be considered.
